# A Glove-Based Form Factor for Collecting Joint Acoustic Emissions: Design and Validation

**DOI:** 10.3390/s19122683

**Published:** 2019-06-13

**Authors:** Nicholas B. Bolus, Hyeon Ki Jeong, Daniel C. Whittingslow, Omer T. Inan

**Affiliations:** 1 Bioengineering Graduate Program, Georgia Institute of Technology, Atlanta, GA 30332, USA; nbolus@gatech.edu; 2 School of Electrical and Computer Engineering, Georgia Institute of Technology, Atlanta, GA 30313, USA; hjeong39@gatech.edu; 3 Wallace H. Coulter Department of Biomedical Engineering, Georgia Institute of Technology, Atlanta, GA 30332, USA; d.c.whittingslow@emory.edu; 4 School of Medicine, Emory University, Atlanta, GA 30322, USA

**Keywords:** acoustic emissions, joint sounds, glove, wearable sensing, knee joint loading

## Abstract

Sounds produced by the articulation of joints have been shown to contain information characteristic of underlying joint health, morphology, and loading. In this work, we explore the use of a novel form factor for non-invasively acquiring acoustic/vibrational signals from the knee joint: an instrumented glove with a fingertip-mounted accelerometer. We validated the glove-based approach by comparing it to conventional mounting techniques (tape and foam microphone pads) in an experimental framework previously shown to reliably alter healthy knee joint sounds (vertical leg press). Measurements from healthy subjects (N = 11) in this proof-of-concept study demonstrated a highly consistent, monotonic, and significant (*p* < 0.01) increase in low-frequency signal root-mean-squared (RMS) amplitude—a straightforward metric relating to joint grinding loudness—with increasing vertical load across all three techniques. This finding suggests that a glove-based approach is a suitable alternative for collecting joint sounds that eliminates the need for consumables like tape and the interface noise associated with them.

## 1. Introduction

Injuries and chronic disorders affecting joints are pervasive and degrade quality of life for millions of individuals [1,2]. The knee joint, due to its anatomical complexity, role in weight bearing, and high, cyclical exposure to mechanical stress, is particularly susceptible to injury [3]. The current diagnostic standard for acute joint injury and chronic conditions such as osteoarthritis involves a combination of medical imaging, which can be costly and time-intensive, and physical examination, which often relies on subjective evaluations made on the part of either the clinician or the patient. Moreover, these methods are not ideally suited to longitudinal, comprehensive monitoring of joint health, which may benefit recovery.

Accordingly, recent work has demonstrated the viability of using the acoustic emissions produced by joints in motion—in particular, the knee—as an indicator of underlying joint health. McCoy et al. referred to the concept of sensing skin vibrations (i.e., their local accelerations) caused by joint articulation as “vibroarthrography” [4]. These vibrations produce an acoustic response in the surrounding media, which is why the signal is often termed a “joint sound” or “acoustic emission.” Arthro-acoustic techniques have been explored in both clinical [5,6,7] and ambulatory settings [8], using both benchtop and wearable equipment [9]. Results from these studies have demonstrated an ability to discriminate reliably between the acoustic signatures of healthy and impaired joints [6,10], and those of joints under varying mechanical load [11]. The latter study validated the use of a vertical leg press as a reliable paradigm for modifying the acoustic output of a healthy knee, demonstrating a change in the heterogeneity of the joint sound as a function of percent body weight applied.

More recently, our group has begun to explore the use of alternative form factors for collecting joint sounds that would improve the quality and reliability of the measurements and eliminate the need for consumables like tape and adhesive microphone pads, which are the conventional means of mounting acoustic sensors on the skin. Drawing inspiration from manual auscultation, we designed a system in which contact microphones are embedded in a glove and placed at locations of interest around a joint to collect arthro-acoustic data. This approach offers several advantages, including the ability to finely regulate contact pressure at the sensor-to-skin interface (by leveraging the user’s inherent motor control and tactile feedback mechanisms) while removing interface noise caused by adhesive, fabric, or other material interacting with the skin. Additionally, an adhesive-based solution is not ideally suited to applications involving repeated use, such as longitudinal tracking in a home setting. Conversely, a hand-worn system such as the one proposed in this work could be easily and repeatedly administered, and, furthermore, would provide an opportunity for an individual to actively engage in the management of one’s own or a dependent’s care—for example, a parent might use the glove to collect joint acoustic data on a child suffering from juvenile idiopathic arthritis.

**Figure 1:**
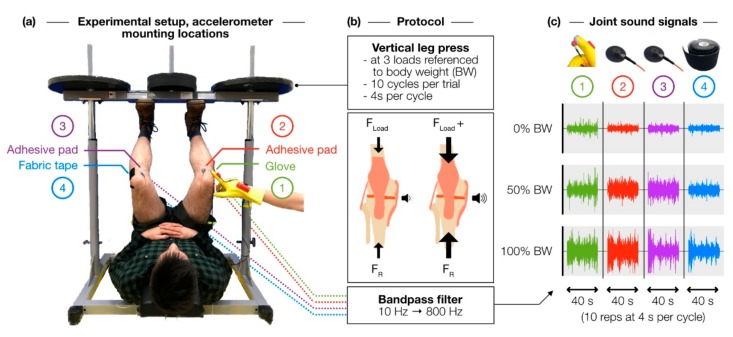
Experiment overview. (**a**) Four accelerometers were placed at regions of interest around the knee joint using different mounting techniques, and, in parallel, recorded vibrations produced by the joint during a vertical leg press exercise. (**b**) Increasing normal forces within the joint, we hypothesized, would increase the loudness of low-frequency grinding sounds within the knee. (**c**) Representative joint sound waveforms demonstrate how the amplitude of low-frequency vibrations increased as a function of percent body weight applied.

In this study, we employed the healthy subjects vertical leg press paradigm (Figure 1) as a means to validate the glove-based approach, alongside two more conventional mounting techniques: fabric tape and adhesive foam mic pads. Achieving a similar result in terms of a quantity that reflects the internal state of the knee joint—i.e., the loudness of grinding—across these techniques would suggest that a glove-based system can provide clinical value comparable to more established techniques without the need for consumables. Multi-day repeatability testing was also conducted to assess the reliability of results derived from the glove-based system, as well as their agreement with results derived from conventional techniques.

## 2. Methods

### 2.1. Design of a Glove-Based Form Factor

**Figure 2:**
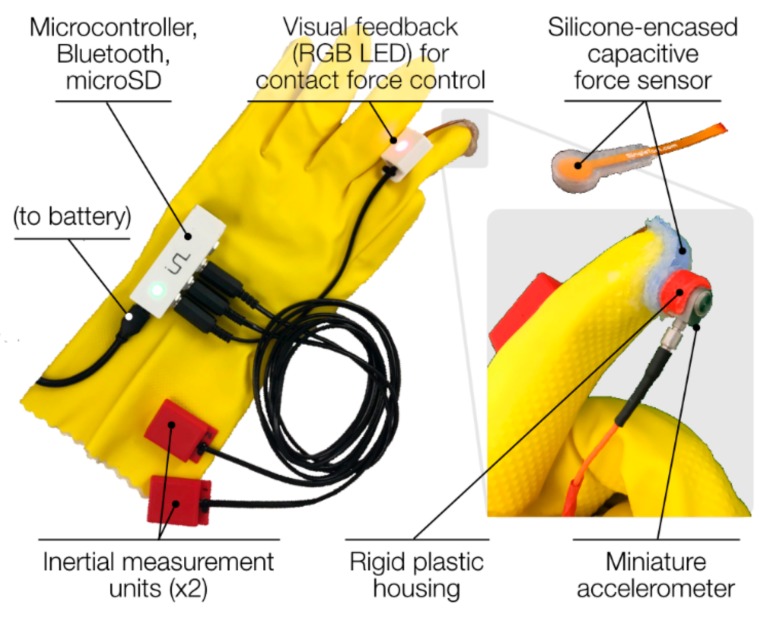
Design of a glove with embedded sensors for capturing joint sounds (accelerometer) and other contextual signals (inertial measurement units for limb motion, capacitive force sensor for sensor–skin contact pressure).

Our glove-based arthro-acoustic sensing system consists of (1) a glove to which various sensing and data acquisition components are mounted, (2) one or more fingertip modules in which the contact accelerometer and force sensor are integrated, and (3) a microcontroller for collecting data and driving feedback mechanisms for fingertip force regulation (Figure 2).

We used a latex/neoprene cleaning glove (Playtex, Dover, DE, USA), because it is easy to disinfect and because its elasticity enables a solid, contoured fit to the user’s digits. Good coupling between the glove/sensors and the hand is critical for maintaining stable contact at the user–subject interface to minimize motion artifacts. 

Sensing of the joint sound signal occurs at the fingertip, where a miniature, high-bandwidth, uniaxial accelerometer (sensitivity = 100 mV/g, frequency response ±10% = 2 to 10,000 Hz) (series 3225, Dytran Instruments, Inc., Chatsworth, CA, USA) is placed in a rigid plastic housing. The accelerometer is sensitive enough to resolve small vibrations caused by the articulation of the internal components of the knee joint that travel to the skin surface [12]. 

**Figure 3:**
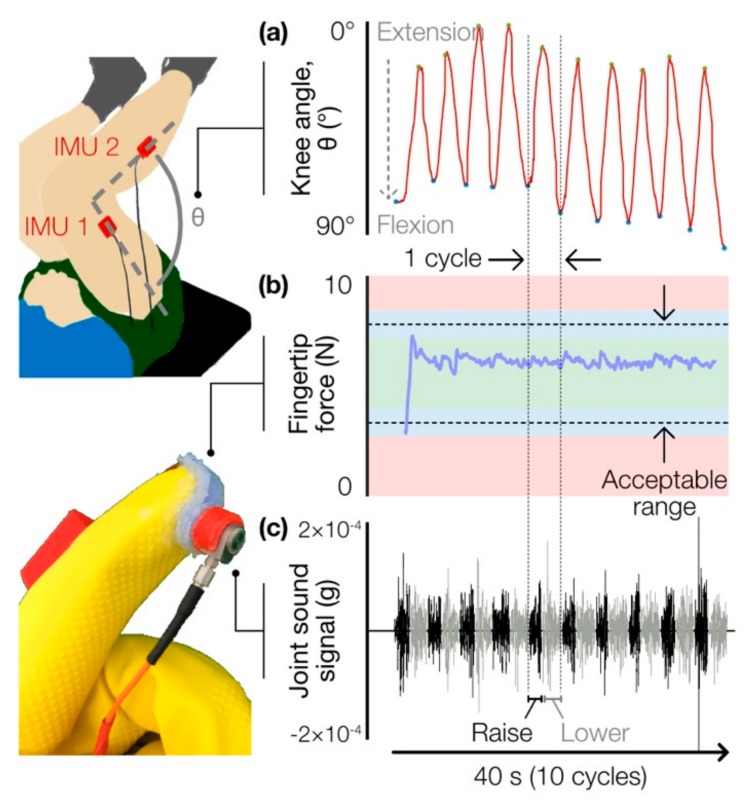
Sample time-series waveforms of signals collected by the glove system during a single experiment trial, consisting of 10 vertical leg press cycles. (**a**) IMUs were used to confirm that consistent knee range of motion (in degrees, °) was achieved at a constant cadence and to segment the joint sound signal into individual cycles. (**b**) Contact force (in N) at the fingertip was measured to confirm a consistent amount of pressure was applied. (**c**) The joint sound signal (local acceleration, in g) was captured by a fingertip-mounted vibration sensor and segmented into cycles consisting of extension (“raise”) and flexion (“lower”) phases.

Sandwiched between the accelerometer and the fingertip is a capacitive force sensor (CS8-10N, SingleTact, Los Angeles, CA, USA) encased in silicone rubber (OOMOO 30, Smooth-On, Lower Macungie, PA, USA). The force sensor (full-scale range = 0–10 N) measures contact pressure between the accelerometer and the subject’s skin. The utility of this measurement is twofold. First, it complements the acoustic signal captured by the accelerometer, providing context such as whether inconsistent contact is made, potentially a source of signal artifact; such context clues can help the researcher gauge the quality of the joint sound recording. Second, the contact force measurement, in conjunction with real-time sensory (e.g., visual, haptic) feedback, can be used as a mechanism for training users to apply consistent pressure at the sensor-to-skin interface, reducing inter-trial and inter-user variability of recordings. In our system, a multi-color LED on the dorsal surface of the index finger provides visual feedback of sensor contact force via an LED color scheme. A green light indicates that the user is pressing within a desired range of contact force for consistent signal acquisition, with light color changing from blue to red as force exits this range (Figure 3b). This feedback mechanism helped ensure that consistent contact pressure was maintained across trials and across subjects. Intermediate values of contact force (roughly between 4 and 7 N) were found to produce repeatable results in terms of root-mean-squared (RMS) amplitude in the frequency band of interest, while pressing too hard (between 8 and 10 N) led to discomfort in some subjects. The current study did not directly assess the effects of contact force on signal properties, which is a limitation of the current approach that will be discussed further. The capacitive force sensor itself, though accurate and reliable, is delicate and prone to delamination, so the custom silicone rubber mold protects the sensor from damage while still allowing it to deflect and measure force. 

Besides sensor–skin interface force, another potentially important variable to account for is the motion of the joint being assessed. To ensure consistent knee joint displacement and velocity—which can affect the acoustic output of the joint [9,13]—across repetitions of the leg press, we integrated two inertial measurement units (IMUs) (BNO055, Bosch Sensortec, Reutlingen, Germany)—one for the shank segment and one for the thigh—into the glove design. These particular sensors are able to perform onboard sensor fusion and thus output a quaternion estimate. These quaternions are used to estimate the knee joint angle across the leg press maneuver.

Data from the capacitive force sensor and both IMUs (Figure 3a,b) were collected by a Teensy 3.6 microcontroller (PJRC, Sherwood, OR, USA) at a sampling rate of 100 Hz and logged on a microSD card. The microcontroller was housed in a custom enclosure, along with a Bluetooth module (SPBT3.0DP1, STMicroelectronics, Geneva, Switzerland) for streaming data to a laptop and sending/receiving a start/stop signal from MATLAB (MathWorks, Natick, MA, USA). A National Instruments data acquisition unit (USB-4432, Austin, TX, USA) was used to collect the acoustic signals from the four accelerometers at 50 kHz per channel.

### 2.2. Loading Experiment Protocol

All human subjects research was conducted under approval from the Georgia Institute of Technology Institutional Review Board. Eleven healthy subjects (seven male/four female, 25.1 ± 2.5 years, 71.4 ± 16.5 kg, 177.5 ± 11.4 cm) with no history of major knee injury were asked to perform a vertical leg press exercise at three loading conditions referenced to body weight (BW)—0% BW, 50% BW, and 100% BW—while the joint sound signals from both knees were recorded simultaneously by four accelerometers—two on each knee. The accelerometer placement scheme is shown in Figure 1a. These locations have been shown to be effective for capturing the vibrations internal to the knee, and, importantly, they are anatomical landmarks that are easy to locate and provide relatively unimpeded (i.e., by muscle and fat) access to the internal joint space [14]. One accelerometer was affixed lateral to the patellar tendon of the left knee using fabric tape (Kinesio Tex, Kinesio, Albuquerque, NM, USA). Two accelerometers were affixed medial to the patellar tendon of both knees using double-sided adhesive foam microphone pads commonly used for skin-mounting lavalier microphones (23 mm Stickie, Rycote, Gloucestershire, UK). These sensors, attached to the corresponding locations on either knee using the same mounting technique, served as a matched comparison to indicate whether a subject’s left and right knee produced disparate results; in this case, a comparison across all four accelerometers would be invalid. Both the fabric tape and microphone pads have been used previously [11,12,14]. Finally, an accelerometer on the index fingertip of the glove was placed against the right knee lateral to the patellar tendon. For consistency, the glove-based acquisition was performed by the same individual for all subjects.

At each loading condition, the subject performed 10 repetitions of the leg press maneuver at a rate of one repetition every 4 seconds (i.e., raise for 2 s, lower for 2 s, repeat). Subjects were asked to traverse the same joint displacement each repetition, which was confirmed visually by marking off upper and lower positions on the leg press machine pylons. Consistent cadence was confirmed by ensuring that the time elapsed between each successive flexion-extension (FE) cycle—i.e., time between local minima of the joint angle estimation—deviated no more than 0.2 s from the ideal 4 s period. Loading conditions were randomized to minimize fatigue and learning effects. Two trials were conducted for each condition to confirm a consistent result.

### 2.3. Signal Processing and Data Analysis

We hypothesized that increasing vertical loading in healthy individuals would cause the articular surfaces in the knee to grind together more forcefully, thus increasing the loudness of the sounds associated with grinding (Figure 1b). By both visual and auditory assessment, we concluded that these grinding sounds were consistent with the lower-amplitude, lower-frequency component of the accelerometer signal. This hypothesis is supported by the fact that other joint sound sensing methods tend to focus on the low-frequency spectrum (<1 kHz) [15]. To that end, we posited that low-pass filtering the signal and then computing its RMS amplitude would give us a reasonable metric for grinding loudness. 

The signals were digitally filtered using a Kaiser-window finite impulse response bandpass filter with bandwidth from 10 to 800 Hz. Frequency content below 10 Hz was removed to account for baseline wander of the accelerometer signal caused by coarse movement of the limb during the leg press task. This filtering approach is distinct from that of other work such as Reference [16], in which the primary goal was to capture large-amplitude, high-bandwidth peaks (“clicks” of the joint) in the acoustic signal. In those studies, air microphones offset from the skin surface were used to record the joint sounds instead of contact microphones placed against tissue; in such a scenario, the low-frequency, low-energy acoustic waves would be greatly attenuated at the skin–air interface, so their contribution was not considered. 

IMU data were used to segment the filtered data into cycles (10 cycles per recording) (Figure 3c), and the signal RMS was computed on a cycle-to-cycle basis. Outlier RMS values (those that were more than three mean absolute deviations from the median) were rejected, and after confirming that the fingertip force and joint range of motion across each cycle were within acceptable ranges (Figure 3a,b), the remaining RMS values were averaged, yielding a single mean RMS value per loading condition for each subject. Each RMS value was normalized to that subject’s baseline RMS (i.e., the RMS value at 0% BW). This allowed for comparison of grinding loudness across subjects while minimizing inter-subject baseline RMS variability.

### 2.4. Repeatability Testing: Protocol and Analysis

#### 2.4.1. Comparison of Repeatability between Mounting Techniques

To determine whether the glove-based joint sound sensing system can produce consistent, repeatable, and reliable measurements from cycle to cycle and from trial to trial, we analyzed the joint sounds from a single subject over three days using intraclass correlation coefficient (ICC). ICC is a widely used technique for assessing the degree of correlation and agreement between measurements [17,18].

The glove-based system and the two conventional mounting techniques (fabric tape and foam microphone pads) were used for comparison. Using each of these techniques, the accelerometer was placed on the medial side of the patella, and the subject was asked to perform five cycles of FE per trial, with three such trials conducted for each mounting technique. The signals were digitally bandpass-filtered (10–800 Hz, same as described above), and several key features commonly used in acoustic analysis were extracted for each FE cycle: acoustic energy, energy entropy, and median normalized frequency of the power spectrum. The data were organized into a matrix in which each row represented a single trial and each column represented a single FE cycle. A total of four datasets were used, three of which exclusively included trials of each of the three mounting techniques, with the fourth dataset containing all trials across all three mounting techniques. The three individual datasets were used to evaluate the internal consistency of each of the mounting techniques, while the combined dataset was used to assess the level of agreement among the three mounting techniques. Using the two-way random effects model [17], ICC values were calculated for each dataset to show the reliability of acoustic features calculated both across FE cycles and across trials.

#### 2.4.2. Effect of Fingertip Contact Force Consistency on Repeatability 

As mentioned previously, a capacitive force sensor embedded in the fingertip of the glove system provides information about sensor-to-skin contact—inconsistent or inadequate contact could produce artifacts in the joint sound signal, and, therefore, unreliable results. To assess the value of the force sensor experimentally, a single subject was asked to perform a seated, unloaded knee FE task while joint sound signals were acquired by a glove-mounted contact microphone at the same mounting position used in the loading experiment described above (i.e., lateral to the patellar tendon of the right knee), along with contact force and IMU data. Testing was performed on a single-subject basis to reduce the contribution of inter-subject variability on results. The subject performed eight trials of unloaded FE at the same cadence as the loading experiment (one repetition every 4 s), with each trial lasting 30 s in total. Across all eight trials, four were conducted under conditions of consistent contact in which the experimenter relied on visual feedback from the RGB LED to modulate fingertip force; the other four trials were conducted under conditions of inconsistent contact, in which the sensor occasionally lost contact with the skin due to imprecise fingertip force control. Repeatability of results was analyzed on a within-trial (i.e., between each FE cycle) and across-trial (i.e., average of all FE cycles from all four trials) basis. Specifically, consistency of the feature of interest, low-frequency RMS amplitude, was quantified using standard deviation (SD) and coefficient of variation (CV)—the ratio of sample standard deviation to sample mean—as metrics of reliability and repeatability.

## 3. Results and Discussion

### 3.1. Effect of Leg Press Load on Knee Grinding Loudness

**Figure 4:**
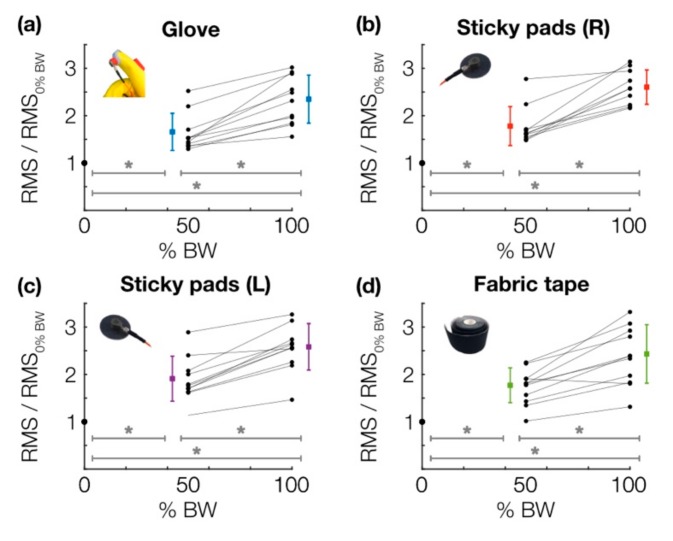
Relative grinding loudness vs. % body weight applied for each mounting technique, including (**a**) the instrumented glove, (**b**) adhesive microphone pads mounted on the right leg, (**c**) adhesive microphone pads mounted on the left leg (for determining comparability between left and right knees), and (**d**) fabric kinesiology tape. Across 11 subjects, each mounting technique demonstrates the same trend: a monotonic, significant increase in baseline-normalized RMS with increasing vertical load. (*) indicates significance (*p* < 0.01) as determined by paired Student’s *t*-test with Holm–Bonferroni correction.

The key result of this study is illustrated in Figure 4, which shows that relative grinding loudness (RMS of the low-pass-filtered joint sound signal, referenced to the no-load, or 0% BW, condition) within the knee increased significantly (*p* < 0.01, using paired sample *t*-test with Holm–Bonferroni correction) and monotonically with vertical loading for all three mounting techniques across subjects. Furthermore, comparison across techniques at each loading condition showed no significant (*p* < 0.01) differences in grinding loudness between the glove, mic pads, and tape. This finding—in particular, that the glove achieved a comparable result (both in terms of the actual RMS quantity and its relationship to the test condition) to that of the other two conventional techniques—supports the idea that a glove-based form factor is an effective approach for capturing and extracting information from joint sounds. 

### 3.2. Repeatability of Glove Versus Conventional Techniques

The central result of repeatability testing is shown in Table 1, which reports the ICC values calculated for each dataset described in Section 2.4.1. While there is no standard value for acceptable reliability using ICC, a general rule suggests that values between 0.5 and 0.75 indicate moderate reliability, values between 0.75 and 0.9 indicate good reliability, and values greater than 0.90 indicate excellent reliability [17]. For the glove-only dataset, repeatability analysis yielded ICC values of 0.984 (95% CI of 0.972–0.992) for the acoustic energy feature, 0.947 (95% CI of 0.905–0.975) for acoustic entropy, and 0.954 (95% CI of 0.916–0.977) for median-normalized frequency of the power spectrum (MDF). These results suggest that, in terms of three features commonly used to describe distinct characteristics of acoustic signals, a glove-based system can acquire consistent and repeatable joint sounds information between FE cycles and across trials. For the tape-only dataset, repeatability analysis yielded ICC values of 0.928 (95% CI of 0.877–0.962) for acoustic energy, 0.735 (95% CI of 0.608–859) for acoustic entropy, and 0.922 (95% CI of 0.867–0.958) for MDF. For the pads-only dataset, repeatability analysis yielded ICC values of 0.937 (95% CI of 0.893–0.967) for acoustic energy, 0.776 (95% CI of 0.608–0.859) for acoustic entropy, and 0.922 (95% CI of 0.867–0.958) for MDF. The results indicate that the level of reliability for pads and tape can be regarded as “good” to “excellent” for acoustic energy and MDF and “moderate” to “good” for acoustic entropy. For the dataset in which all three mounting techniques were included, repeatability analysis yielded ICC values of 0.982 (95% CI of 0.972–0.989) for acoustic energy, 0.836 (95% CI of 0.742–0.903) for acoustic entropy, and 0.976 (95% CI of 0.962–0.986) for MDF. These results demonstrate a high degree of agreement between features derived from each mounting technique, which further suggests that the glove-based system is a reliable alternative to the conventional methods of mounting the acoustic/vibration sensors to skin.

### 3.3. Consistent Contact Force Improves Consistency of Results

**Figure 5:**
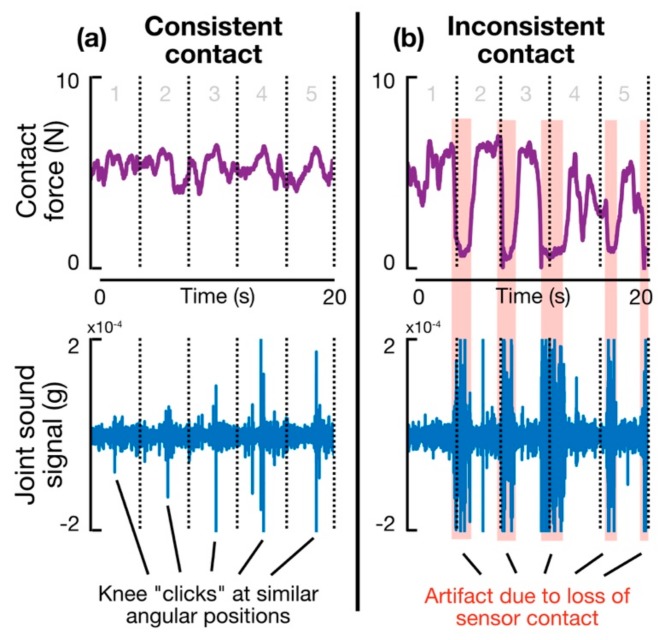
Fingertip contact force and joint sound signal waveforms for representative trials with (**a**) consistent and (**b**) inconsistent sensor–skin contact. Time duration of each waveform is 20 s, in which five seated knee flexion–extension cycles were completed at a rate of 4 s per cycle. Highlighted portions illustrate how a rapid decrease in contact force coincides with regions of the joint sound signal dominated by artifact. These data demonstrate that sensor-to-skin contact force can be used as a context clue for rejection of noisy, low-quality joint sound signals.

Figure 5 shows a snippet of two representative trials comparing the effects of consistent versus inconsistent contact force on the joint sound signal captured by a fingertip-mounted accelerometer. Figure 5b depicts how loss of sensor contact (characterized by a rapid decrease in the contact force signal) coincides with regions of the joint sound signal corrupted by signal artifact. Importantly, these signals serve as an example of how the capacitive force sensor can be used to identify unreliable or low-SNR portions of a joint sound recording. Table 2 demonstrates the benefit of consistent contact on the acquired joint sound signal more quantitatively. In this table, values of mean, standard deviation, and coefficient of variation of “grinding loudness” (low-frequency RMS amplitude) are reported for each FE trial and across trials for both test conditions (i.e., consistent and inconsistent force applied at the fingertip). The key takeaway can be found in the last column of the table, in which the variation in grinding loudness across all FE cycles collected with consistent contact (CV = 0.131) can be seen to be, on average, less than 25% that of the trials with inconsistent contact (CV = 0.550). This finding highlights the fact that consistent contact is critical for obtaining reliable results. 

### 3.4. Considerations for a Hand-Worn Acoustic Sensing System

Using a glove-based system to measure joint sounds presents both benefits and challenges. This technique offers better sensor-to-skin contact, given the nervous system’s capacity for precise endpoint control, but introduces the possibility of user error. The mechanical sensitivity required to resolve vibrations on the surface of the skin caused by internal motion/friction of the joint makes the job of the glove wearer that much more difficult, for a slight change in fingertip contact with the subject’s skin can corrupt the underlying joint sound signal. Thus, contact force feedback and training are important to minimize human error. Other techniques such as tape do not suffer the same limitation, but they have their own. Namely, any approach that uses adhesive has the potential to couple interface sounds—e.g., tape lifting on/off of the skin—into the recording. Furthermore, these artifacts can be difficult to distinguish from the joint sound signal or can bury it entirely. In this way, a glove-based system, coupled with some feedback mechanism and adequate training, presents a major advantage over other, more established techniques. Additionally, a glove-based system eliminates the need for disposables like sticky pads or tape, the use of which may cause discomfort and of which the adhesive may degrade during use, leading to inconsistency in sensor-to-skin contact. Furthermore, the benefit of a glove form factor over a wearable brace with embedded sensors is its versatility of use across joints and across subjects of different sizes/shapes; a brace would require custom fitting—a potentially painstaking task to ensure optimal, consistent contact.

## 4. Conclusions and Future Work

The work reported here—in which we used an experimental framework known to reliably alter healthy knee joint sounds, coupled with a simple metric (low-frequency RMS amplitude) that manifests some changing physical properties of the joint—serves chiefly as a proof-of-concept validation of our glove-based method of joint sound sensing. Validation was further conducted through repeatability testing, which indicated that our glove-based system was able to produce consistent results, particularly under conditions of consistent fingertip contact force, and a high level of agreement with conventional techniques used to couple vibration sensors to skin. Future efforts should focus on identifying additional metrics for comparing the utility/performance of various form factors and, more importantly, deploying the glove in affected populations (e.g., acute knee injury, arthritis). Inter-user variability should be studied to establish the repeatability of results when different users administer the glove. As mentioned previously, this variability may arise from a host of factors, including accuracy of sensor placement near the targeted anatomical landmark and amount of contact pressure applied. While these are limitations of the current approach, future efforts will focus on evaluating the effects of these variables experimentally. We believe that training (i.e., by a medical professional, user manual, or on-device sensory feedback) and experience will be critical for obtaining reliable results, as is the case with any self-administered medical exam or intervention. We envision that a wearable, hand-worn system, when used in a home setting, could serve not only as an effective tool for capturing arthro-acoustic information but also as an opportunity for an individual to directly partake in the healthcare of a dependent, such as the parent of a child with juvenile idiopathic arthritis. Whatever the technique or application, as the value of a joint-sounds-based approach to joint health assessment is better developed, exploration of different techniques and improvements on existing ones will be critical for obtaining the best possible information and achieving the best possible clinical outcome. 

## Figures and Tables

**Table 1:** Results of repeatability testing using intraclass correlation coefficient (ICC) as an indicator of measurement repeatability and agreement among mounting techniques.

	Glove		Tape		Sticky Pads		Glove, Tape, and Sticky Pads	
	ICC	95% CI *	ICC	95% CI *	ICC	95% CI *	ICC	95% CI *
Acoustic Energy	0.984	0.972–0.992	0.928	0.877–0.962	0.937	0.893–0.967	0.982	0.972–0.989
Acoustic Entropy	0.947	0.905–0.975	0.735	0.648–0.859	0.776	0.672–0.871	0.836	0.742–0.903
Median Frequency Power	0.954	0.916–0.977	0.922	0.867–0.958	0.921	0.865–0.959	0.976	0.962–0.986

* CI = Confidence Interval.

**Table 2:** Effects of contact force consistency on repeatability statistics of “grinding loudness” (low-frequency root-mean-square (RMS) amplitude) feature.

		Within Trial (Cycle-to-Cycle)			Across Trials		
		Mean	SD *	CV **	Mean	SD *	CV **
**Consistent contact**	trial 1	8.98 × 10^−3^	7.10 × 10^−4^	0.079	1.02 × 10^−2^	1.34 × 10^−3^	**0.131**
	trial 2	9.63 × 10^−3^	1.56 × 10^−3^	0.162			
	trial 3	1.09 × 10^−2^	7.64 × 10^−4^	0.070			
	trial 4	1.14 × 10^−2^	4.13 × 10^−4^	0.036			
**Inconsistent contact**	trial 1	1.93 × 10^−2^	5.22 × 10^−3^	0.270	3.24 × 10^−2^	1.78 × 10^−2^	**0.550**
	trial 2	2.32 × 10^−2^	7.07 × 10^−3^	0.305			
	trial 3	4.58 × 10^−2^	2.15 × 10^−2^	0.469			
	trial 4	4.11 × 10^−2^	1.73 × 10^−2^	0.422			

* SD = standard deviation, ** CV = coefficient of variation.
